# Supplementary material for: Causal Relationship between Aging and Anorexia Nervosa: A White-Matter-Microstructure-Mediated Mendelian Randomization Analysis
Source: Biomedicines. 2024 Aug 16;12(8):1874. doi: 10.3390/biomedicines12081874 (PMC11351342; doi:10.3390/biomedicines12081874)
Supplement: Supplementary file 1 [file biomedicines-12-01874-s001.zip › Supplementary Figure/Figure S4/Singe SNP plot of MR analysis for TL to ubm-b-1925.pdf]

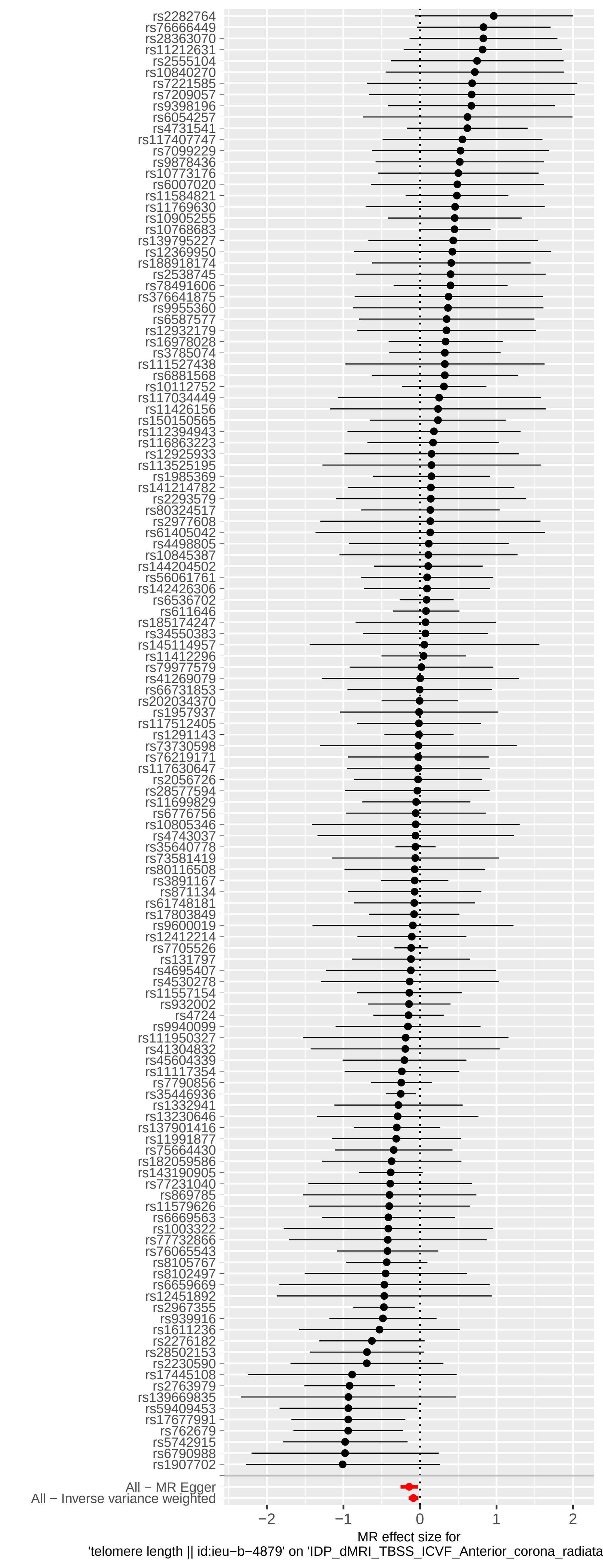
rs2282764
rs76666449
rs28363070
rs11212631
rs2555104
rs10840270
rs7221585
rs7209057
rs9398196
rs6054257
rs4731541
rs117407747
rs7099229
rs9878436
rs10773176
rs6007020
rs11584821
rs11769630
rs10905255
rs10768683
rs139795227
rs12369950
rs188918174
rs2538745
rs78491606
rs376641875
rs9955360
rs6587577
rs12932179
rs16978028
rs3785074
rs111527438
rs6881568
rs10112752
rs117034449
rs11426156
rs150150565
rs112394943
rs116863223
rs12925933
rs113525195
rs1985369
rs141214782
rs2293579
rs80324517
rs2977608
rs61405042
rs4498805
rs10845387
rs144204502
rs56061761
rs142426306
rs6536702
rs611646
rs185174247
rs34550383
rs145114957
rs11412296
rs79977579
rs41269079
rs66731853
rs202034370
rs1957937
rs117512405
rs1291143
rs73730598
rs76219171
rs117630647
rs2056726
rs28577594
rs11699829
rs6776756
rs10805346
rs4743037
rs35640778
rs73581419
rs80116508
rs3891167
rs871134
rs61748181
rs17803849
rs9600019
rs12412214
rs7705526
rs131797
rs4695407
rs4530278
rs11557154
rs932002
rs4724
rs9940099
rs111950327
rs41304832
rs45604339
rs11117354
rs7790856
rs35446936
rs1332941
rs13230646
rs137901416
rs11991877
rs75664430
rs182059586
rs143190905
rs77231040
rs869785
rs11579626
rs6669563
rs1003322
rs77732866
rs76065543
rs8105767
rs8102497
rs6659669
rs12451892
rs2967355
rs939916
rs1611236
rs2276182
rs28502153
rs2230590
rs17445108
rs2763979
rs139669835
rs59409453
rs17677991
rs762679
rs5742915
rs6790988
rs1907702
All − MR Egger
All − Inverse variance weighted
−2
−1
0
1
2
MR effect size for
'telomere length || id:ieu−b−4879' on 'IDP_dMRI_TBSS_ICVF_Anterior_corona_radiata
